# Genome sequences of isolates from high-touch surfaces in washrooms at a post-secondary institution

**DOI:** 10.1128/MRA.00910-23

**Published:** 2023-11-16

**Authors:** Devin B. Holman, Katherine E. James-Gzyl, Rachael L. Rieberger, Kiya S. Keim, Michael J. Carson, Sean G. Norris, Ahmad Esmaeili Taheri

**Affiliations:** 1 Lacombe Research and Development Centre, Agriculture and Agri-Food Canada, Lacombe, Alberta, Canada; 2 Donald School of Business, Science, and Technology, Red Deer Polytechnic, Red Deer, Alberta, Canada; Indiana University, Bloomington, Indiana, USA

**Keywords:** *Brevibacterium casei*, *Heyndrickxia oleronia*, *Kocuria palustris*, *Microbacterium *spp., *Staphylococcus cohnii*, *Staphylococcus epidermidis*, genome sequencing, high-touch surfaces

## Abstract

We report here the draft genome sequences of *Brevibacterium casei* (*n* = 1), *Heyndrickxia oleronia* (*n* = 1), *Kocuria palustris* (*n* =1), *Microbacterium* spp. (*n* = 5), *Staphylococcus cohnii* (*n* = 3), and *Staphylococcus epidermidis* isolated from high-touch surfaces in washrooms at a post-secondary institution.

## ANNOUNCEMENT

High-touch objects (paper towel dispenser handle, soap dispenser handle, and toilet seat) in public washrooms on the Red Deer Polytechnic main campus in Red Deer, Alberta, Canada, were swabbed and streaked onto CHROMagar MRSA (methicillin-resistant *Staphylococcus aureus*) (CHROMagar, Paris, France) with the objective of detecting MRSA or other methicillin-resistant *Staphylococcus* spp. MRSA is a potential pathogen that can cause serious infections in both healthcare and community settings (1). CHROMagar MRSA plates were incubated at 37°C for 48 h, and one mauve colony per plate was then selected, re-streaked onto brain heart infusion (BHI) agar, and incubated at 37°C for 48 h. The resulting 12 isolates were re-grown in 10 mL broth BHI overnight at 37°C, and 1.5 mL was pelleted and DNA extracted with the DNeasy Blood and Tissue kit (Qiagen, Toronto, ON, Canada) as described by the manufacturer. The genomes of these isolates were then sequenced as follows. The DNA concentration was assessed with a Qubit dsDNA HS Assay Kit (Thermo Fisher Scientific, Mississauga, ON, Canada), and 500 ng was used as input with the Illumina DNA Prep Kit (Illumina Inc., San Diego, CA, USA) to prepare genomic libraries. Each library was then diluted to 4 nM and pooled. Prior to loading, the pooled library was denatured and diluted to a final loading concentration of 10 pM according to the supplier’s protocol and denatured PhiX (1%) (Illumina Inc.) was added to the library. The libraries were sequenced on a MiSeq instrument using the MiSeq Reagent Kit v2 (2 × 150 bp; Illumina Inc.).

Sequences were quality filtered using fastp v. 0.23.2 with automatic adapter trimming, and reads were discarded if they were less than 100 bp in length or had a mean Phred quality score below 15 within a sliding window of 4 bp. SPAdes v.3.15.5 (2) with the “isolate” option was used to assemble the paired-end reads, and assembly quality was assessed with QUAST v.5.0.2 (3). The Genome Taxonomy Database Toolkit (GTDB-tk) v. 2.2.4 (4) with GTDB release 207 (5) was used to classify the genome assemblies. Assemblies were annotated by NCBI using the prokaryotic genome annotation pipeline (PGAP) 2022-10-03.build6384 (6). Antimicrobial resistance genes were identified using the Comprehensive Antibiotic Resistance Database v. 3.2.5 with the Resistance Gene Identifier v. 6.0.1 (7). The average nucleotide identity (ANI) between strains was calculated using fastANI v.1.33 (8). Default parameters were used for all software unless otherwise specified, and the assembly and sequencing characteristics for each genome are shown in Table 1. Although all isolates grew on CHROMagar MRSA, none were identified as *S. aureus* after genome sequencing. However, all *Staphylococcus cohnii* (*n* = 3) and *Staphylococcus epidermidis* (*n* = 1) genomes carried the *mecA* gene for methicillin resistance which may explain their selection on CHROMagar MRSA. Based on their ANI values (>99%), the three all *S. cohnii* isolates were from the same strain as were the five *Microbacterium* spp. isolates. The *Microbacterium* spp. isolates appeared to belong to a potentially novel species based on their ANI to other *Microbacterium* spp. genomes in the GTDB.

**TABLE 1:** Isolate and sequence summary^*a*^

BioSample accession no.	Isolate ID	Species	Source	Genome accession no.	SRA accession no.	No. of contigs	No. of reads	Genome size (bp)	*N*_50_ value (bp)	Avg coverage (×)	No. of coding sequences	G + C content (%)	ARGs
SAMN33819725	RD01	*Staphylococcus cohnii*	Paper towel dispenser	JAROYR000000000	SRR24288510	98	1924778	2739586	139583	105	2,674	32.4	*mecA*, *mecI*, *mecR1*, *mph*(C), *msr*(A)
SAMN33819726	RD02	*Microbacterium* sp. RD02	Soap dispenser	JAROYQ000000000	SRR24288509	128	1055180	3687562	45298	43	3,666	69.8	
SAMN33819727	RD03	*Heyndrickxia oleronia*	Soap dispenser	JAROYP000000000	SRR24288506	46	1593812	5398352	248092	44	5,321	35.0	*blaI, msr*(G)
SAMN33819728	RD04	*Staphylococcus cohnii*	Paper towel dispenser	JAROYO000000000	SRR24288505	78	1240138	2713842	143395	69	2,650	32.3	*mecA*, *mecI*, *mecR1*, *mph*(C), *msr*(A)
SAMN33819729	RD05	*Kocuria palustris*	Soap dispenser	JAROYN000000000	SRR24288504	141	1369204	2957926	36101	69	2,696	70.1	
SAMN33819730	RD06	*Microbacterium* sp. RD06	Paper towel dispenser	JAROYM000000000	SRR24288503	79	1255876	3686809	108078	51	3,633	69.8	
SAMN33819731	RD07	*Staphylococcus epidermidis*	Paper towel dispenser	JAROYL000000000	SRR24288502	47	2907210	2455217	100095	178	2,292	32.0	*ant(4')-Ib, blaZ, mecA*,
SAMN33819732	RD08	*Brevibacterium casei*	Paper towel dispenser	JAROYK000000000	SRR24288501	74	1094536	3798349	121230	43	3,426	68.2	
SAMN33819733	RD09	*Staphylococcus cohnii*	Paper towel dispenser	JAROYJ000000000	SRR24288500	82	1342708	2717720	143395	74	2,650	32.3	*mecA*, *mecI*, *mecR1*, *mph*(C), *msr*(A)
SAMN33819734	RD10	*Microbacterium* sp. RD10	Toilet seat	JAROYI000000000	SRR24288499	79	1330330	3686251	99937	54	3,632	69.8	
SAMN33819735	RD11	*Microbacterium* sp. RD11	Paper towel dispenser	JAROYH000000000	SRR24288508	80	1247482	3668059	97322	51	3,609	69.8	
SAMN33819736	RD12	*Microbacterium* sp. RD12	Paper towel dispenser	JAROYG000000000	SRR24288507	127	1061090	3688021	52461	43	3,658	69.8	

^*a*^ ARGs, antimicrobial resistance genes.

## Data Availability

All raw genome sequences and draft genome assemblies have been deposited in the Sequence Read Archive and GenBank, respectively, under the accession numbers listed in Table 1.
